# *In Situ* Roughness Measurements for the Solar Cell Industry Using an Atomic Force Microscope

**DOI:** 10.3390/s100404002

**Published:** 2010-04-20

**Authors:** Higinio González-Jorge, Victor Alvarez-Valado, Jose Luis Valencia, Soledad Torres

**Affiliations:** 1 Departamento de Ingeniería de los Recursos Naturales y del Medioambiente, ETS Ingeniería de Minas, Universidad de Vigo, 36310 Vigo, Spain; 2 Departamento de I+D, Laboratorio Oficial de Metroloxía de Galicia, Parque Tecnolóxico de Galicia, San Cibrao das Viñas, 32901 Ourense, Spain; E-Mails: valvarez@lomg.net (V.A-V.); jlvalencia@lomg.net (J.L.V.); storres@lomg.net (S.T.)

**Keywords:** surface metrology, atomic force microscopy, solar cell, transparent conductive oxide, areal roughness

## Abstract

Areal roughness parameters always need to be under control in the thin film solar cell industry because of their close relationship with the electrical efficiency of the cells. In this work, these parameters are evaluated for measurements carried out in a typical fabrication area for this industry. Measurements are made using a portable atomic force microscope on the CNC diamond cutting machine where an initial sample of transparent conductive oxide is cut into four pieces. The method is validated by making a comparison between the parameters obtained in this process and in the laboratory under optimal conditions. Areal roughness parameters and Fourier Spectral Analysis of the data show good compatibility and open the possibility to use this type of measurement instrument to perform *in situ* quality control. This procedure gives a sample for evaluation without destroying any of the transparent conductive oxide; in this way 100% of the production can be tested, so improving the measurement time and rate of production.

## 1. Introduction

Roughness parameters describe the irregularities that occur due to the material removal processes, including tool geometry, wheel grit, electrical discharge machining spark, and chemical or physical vapor deposition [1–3]. In this sense, roughness parameters are key factors for a number of industrial manufactured products and directly influence their subsequent performance. Some examples are the relationship of roughness with tribology [4,5], the reflectivity of optical products fabricated by diamond turning [6] or moulding [7] and the adhesion of paint and plating applications [8].

Areal surface evaluation is of greater interest than 2D profiles. For example, if a random profile is taken from an areal surface measurement, differences between discrete pits and scratches are not revealed; for this reason the areal image is needed. Restriction in areal surface measurements comes from the range and resolution of the instrumentation available, although several kinds of measuring equipment are available for related applications (*i.e.*, atomic force microscopy, optical or stylus profilometry) [9].

Atomic force microscopes (AFMs) are especially useful when high lateral resolution is required and the measurement range is small [10]. One of the typical problems relating to this technology is the necessity to destroy the manufactured part to obtain an evaluation sample, which is moved from the fabrication area to the laboratory area, where the AFM is usually located. Recently some AFM manufactures have provided instruments that are portable and therefore can, at first sight, be used to make *in situ* measurements in industry [11,12]. Moreover, a lot of work must be performed to demonstrate their real behavior in each specific industrial environment.

The thin film photovoltaic industry has been growing significantly over recent years, driven by the increasing demand of photovoltaic power plants and the higher price of silicon. The front panel of this kind of solar cell consists of a transparent conductive oxide (TCO) material which allows light transmission and also serves as an electric contact. TCO roughness is an important parameter in quality control because it is directly related to the electrical efficiency of the module [13,14]. This parameter is nowadays usually checked using mechanical profilers (2D roughness) or AFMs (areal roughness). This study is carried out using an evaluation sample which is taken from a TCO panel removed from the fabrication area. The problems for this method are time consumption, destruction of the TCO panel and the impossibility to make a control, at least on a small area, in 100% of production.

In this work, we make a metrological comparison between the areal roughness, determined by an AFM measurement on a TCO panel, in both the fabrication and laboratory areas of a solar cell company. The aim of this feasibility study is to demonstrate the *in situ* capability of using this instrument for quality control and open the possibility of extending its use.

## 2. Experimental

### 2.1. AFM Instrument

**Figure 1.**
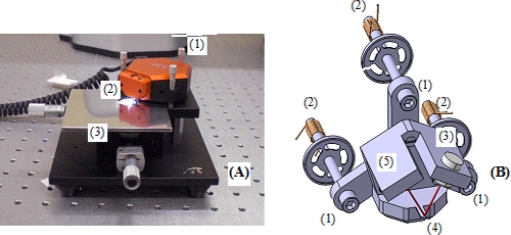
(A) AFM Nanosurf Easyscan 2 and (B) scene of the scanning tripod.

The AFM used in these experiments is the Nanosurf Easyscan 2 (Figure 1). This AFM head is different to those normally used, which are based on piezoelectric tube scanners, and its motion and measurement principles are described below.

The coarse height of AFM is provided by three large vertical screws (Figure 1-A1) at the edges of the unit and this traditional mechanical arrangement is complemented with a motorized movement (Figure 1-A2) that allows the cantilever tip to approach the sample safely. Fine scale x-y-z movement of the cantilever is controlled from inside the head assembly (Figure 1-B). A tripod is suspended on three flat shaped springs with three iron cylinders (Figure 1-B1) behind them, each spaced a little in front of small electromagnets (Figure 1-B2). Different energy supplied to the electromagnets produces different cantilever motions. For instance, when all the electromagnets are energized together, the cantilever moves straight back. On the other hand, if the right electromagnet is energized the cantilever moves right.

The measurement principle is the same as in traditional AFMs. It consists of the combination of a laser diode (Figure 1-B3), which generates the beam that irradiates the back of the cantilever tip (Figure 1-B4), with the reflected beam analyzed by a photodetector (Figure 1-B5).

It should be noted that the portable concept has been achieved because the measurement and the motion system are both assembled in the same AFM head. AFM instruments, where the motion actuator is in the horizontal stage and the measurement system is in the head, cannot be used for portable measurements.

The horizontal stage (Figure 1-A3) of the AFM can be only used for the measurements made in the company laboratory and not when it is acting as a portable instrument in the fabrication area.

The measurement parameters selected for these experiments are as follows:

- Operating mode: Tapping mode
- Cantilever force: 20 nN
- Image resolution: 256 points per line (ppl)
- Lines: 256
- Time per line: 1 s
- Cantilever type: Nanosensors NCLR [15]
- Vibration frequency: 159.4 kHz
- Measurement time: 8 min 30 s (approximate)

### 2.2. Validation Process

**Figure 2.**
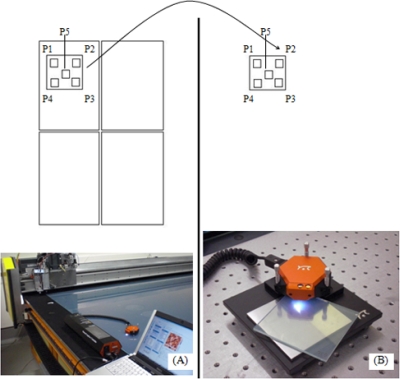
Measurement configurations in five sub-topographies (A) *in situ* and (B) in laboratory. P1–P5 indicate the regions where the sub-topographies were made.

The fabrication area selected for performance of the measurements is on the cutting table because this place is relatively isolated from acoustic noise. The cutting table is based on a CNC diamond cutting machine which obtains four TCO panels of 1.35 m^2^ from the larger one of 5.4 m^2^. Nowadays areal roughness of the panels is only measured in a small number of them. This quality control process is performed cutting a small part of the panel which is then scanned with the AFM in the laboratory. This panel is then removed from the production line and destroyed. In this paper we try to validate the AFM possibility to perform *in situ* measurements without destroying the panel, so we establish the following steps:

- A region of interest (10 cm^2^) is selected on a large panel (5.4 m^2^) on the cutting table (Figure 2).
- Five sub-topographies are obtained from this region using the AFM in portable configuration.
- The region of interest is cut and moved to the laboratory. The laboratory room is isolated from noise and vibration and the AFM is placed on an optical table with pneumatic isoltation.
- Sample in the laboratory is measured and roughness results are compared with those previously obtained to demonstrate the possibility or not to use the AFM as portable instrument in the fabrication of a solar cell industry.

The areal roughness parameters to be compared for the two measurement configurations are roughness average Sa and roughness root mean square Sq [16]. Data evaluation is performed for all of the 65,536 topography points. Mean values and standard deviation are obtained for both fabrication and laboratory measurements, for comparison. In addition, all data were processed using a Fast Fourier Transform to make a spectral analysis and compare noise influences between both configurations.

## 3. Results and Discussion

**Figure 3.**
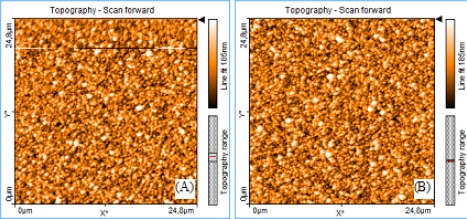
Top left sub-topographies—P1 obtained (A) *in situ* and (B) in laboratory.

**Figure 4.**
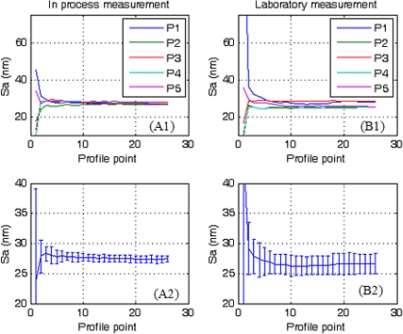
Results for areal roughness parameter Sa (A) *in situ* and (B) in laboratory. Index 1 represents the raw data and 2 the average of the five sub-profiles. Error bars indicate the standard deviation of the average.

**Figure 5.**
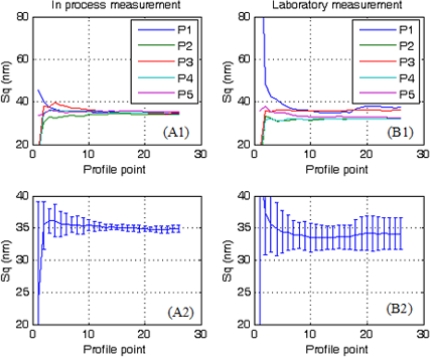
Results for areal roughness parameter Sq (A) *in situ* and (B) in laboratory. Index 1 represents the raw data and 2 the average of the five sub-profiles. Error bars indicate the standard deviation of the average.

Figure 3 shows the top left sub-topographies (P1 in Figure 2) obtained for the fabrication and laboratory areas. Figures 4 and 5 depict the evaluation of the areal roughness parameters Sa and Sq, respectively. Table 1 exhibits the values and standard deviation obtained for the areal roughness average and root mean square from the five sub-topographies in the two areas of study. Each profile point represents the contribution of 8 lines of the profile (2,048 topography data with 256 ppl). Only these values where represented on the plots to avoid overcharging of the computer memory.

Figures 4 and 5 show a high variability in the roughness parameters during the first profile points, but they tend to converge to one asymptotic value over the fifth profile point (10,240 topography data involved in the calculation). This is due to the fact that roughness is a statistical parameter and a high number of input data are needed to obtain a good result.

Comparison between the roughness parameters (Table 1) reveals good agreement between the fabrication and laboratory areas, demonstrating that *in situ* measurements can be performed with this portable AFM. Although the set of samples are the same under the two measurement procedures, it is remarkable that higher standard deviation is obtained from the values in the laboratory area. As environmental conditions are better in laboratory than in factory, the reason for these results are the differences of intrinsically roughness values obtained from the sub-topographies. For example in Figure 4-B1 deviation in laboratory come from the roughness value differences between sub-topographies P1–P5 and not for self topography deviations. Such results are in agreement with the roughness tolerance of the panel (4 nm—manufacturer data), so this source of error can only arise from the sample non-uniformity and not from instabilities in the measurement procedure or tip artifacts. Previous experiments made for our group show as changes in roughness measurements using different tips are under the tolerance of the own TCO material

**Figure 6.**
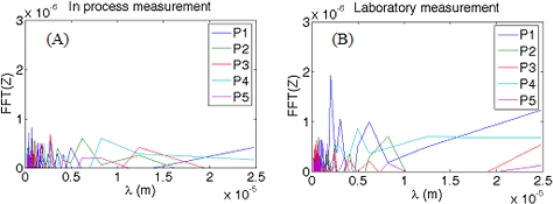
Fourier Spectral Analysis for measurements performed (A) *in situ* and (B) in laboratory.

To complete the study a Fourier Spectral Analysis [1] was made for all the sub-topographies (Figure 6). Both situations show a similar pattern, which demonstrates a similar influence from small wavelength noise (typically from sound pressure level or vibrations) or long wavelength noise (typically from temperature fluctuations).

## 4. Conclusions

This research work shows a comparative study between several parameters obtained using an AFM acting as a portable instrument in the fabrication area of the solar cell company and placed in the laboratory area, where tip influence is not relevant for the rough TCO samples. Results obtained in both situations are in agreement and they show the capability of using this instrument for *in situ* measurements. This new configuration would reduce the time required for quality control, because the sample does not have to be moved to the laboratory and avoids the need to destroy the TCO panel to obtain an evaluation sample, 100% of the production output may be inspected. This study tries to be a first approach to open the possibility to use this kind of instrumentation under fabrication conditions. Much work must be make for the future as for example to study the behavior of different AFM instruments or study how the hard working conditions affect to AFM metrological stability.

## Figures and Tables

**Table 1.** Areal values and standard deviation of roughness average and roughness root mean square obtained from the five sub-topographies in the fabrication and laboratory areas.

	**Fabrication area**	**Laboratory area**
***Sa* (nm)**	27.4 ± 0.6	26.6 ± 1.7
***Sq* (nm)**	34.9 ± 0.6	34.1 ± 2.4
